# Integrative Metabolomic and Transcriptomic Analyses Reveal the Mechanism of Petal Blotch Formation in *Rosa persica*

**DOI:** 10.3390/ijms25074030

**Published:** 2024-04-04

**Authors:** Huan Wang, Ying Kong, Xiaoying Dou, Yi Yang, Xiufeng Chi, Lixin Lang, Qixiang Zhang, Huitang Pan, Jinrong Bai

**Affiliations:** 1 State Key Laboratory of Efficient Production of Forest Resources, Beijing Key Laboratory of Ornamental Plants Germplasm Innovation & Molecular Breeding, National Engineering Research Center for Floriculture, Beijing Laboratory of Urban and Rural Ecological Environment, School of Landscape Architecture, Beijing Forestry University, Beijing 100083, China; wanghuan@bjfu.edu.cn (H.W.); yiyang@bjfu.edu.cn (Y.Y.); chixiufeng1203@bjfu.edu.cn (X.C.); zqx@bjfu.edu.cn (Q.Z.); 2 Institute of Radiation Technology, Beijing Academy of Science and Technology, Beijing 100875, China; kongying@bjast.ac.cn (Y.K.); douxiaoying@bjast.ac.cn (X.D.); langlixin@bjast.ac.cn (L.L.)

**Keywords:** flower color pattern, metabolome, transcriptome, WGCNA, differentially expressed genes

## Abstract

Petal blotch is a specific flower color pattern commonly found in angiosperm families. In particular, *Rosa persica* is characterized by dark red blotches at the base of yellow petals. Modern rose cultivars with blotches inherited the blotch trait from *R. persica*. Therefore, understanding the mechanism for blotch formation is crucial for breeding rose cultivars with various color patterns. In this study, the metabolites and genes responsible for the blotch formation in *R. persica* were identified for the first time through metabolomic and transcriptomic analyses using LC-MS/MS and RNA-seq. A total of 157 flavonoids were identified, with 7 anthocyanins as the major flavonoids, namely, cyanidin 3-*O*-(6″-*O*-malonyl) glucoside 5-*O*-glucoside, cyanidin-3-*O*-glucoside, cyanidin 3-*O*-galactoside, cyanidin *O*-rutinoside-*O*-malonylglucoside, pelargonidin 3-*O*-glucoside, pelargonidin 3,5-*O*-diglucoside, and peonidin *O*-rutinoside-*O*-malonylglucoside, contributing to pigmentation and color darkening in the blotch parts of *R. persica*, whereas carotenoids predominantly influenced the color formation of non-blotch parts. Zeaxanthin and antheraxanthin mainly contributed to the yellow color formation of petals at the semi-open and full bloom stages. The expression levels of two 4-coumarate: CoA ligase genes (Rbe014123 and Rbe028518), the dihydroflavonol 4-reductase gene (Rbe013916), the anthocyanidin synthase gene (Rbe016466), and UDP-flavonoid glucosyltransferase gene (Rbe026328) indicated that they might be the key structural genes affecting the formation and color of petal blotch. Correlation analysis combined with weighted gene co-expression network analysis (WGCNA) further characterized 10 transcription factors (TFs). These TFs might participate in the regulation of anthocyanin accumulation in the blotch parts of petals by modulating one or more structural genes. Our results elucidate the compounds and molecular mechanisms underlying petal blotch formation in *R. persica* and provide valuable candidate genes for the future genetic improvement of rose cultivars with novel flower color patterns.

## 1. Introduction

Flower color patterns, such as stripes, spots, and blotches, are widely distributed in angiosperms. These patterns are of great importance for plants during pollination, defense, and environmental changes [1,2,3,4]. Furthermore, they enrich the color range of flowers and greatly increase the plant’s ornamental and commercial value. Roses are renowned ornamental plants and are widely cultivated around the world. More than 30,000 modern roses are used worldwide [5], with a variety of flower colors. Notably, modern rose cultivars with floral blotches have a unique color phenotype that enhances their ornamental value. In a recent study on the rose cultivar ‘Sunset Babylon Eyes’ only cyanidin 3,5-*O*-diglucoside was found to contribute to blotch formation, while flavonol glycosides acted as yellow background colors and carotenoids made the petal color bright. Moreover, three enzyme genes (*F3′H*, *DFR*, and *ANS*) and two TFs (WRKY and MYB) were identified that may activate anthocyanin accumulation in blotch parts [6]. These findings served as a reference for the blotch formation studies. However, no other research on the blotch formation of roses has been reported.

The diversity of flower color patterns is mainly determined by the differential accumulation of pigments [3]. Flavonoids, carotenoids, and betalains are the three main types of pigments responsible for flower color [7]. The first two pigments are widely distributed across flowering plants. Flavonoids, especially anthocyanins, are the most important pigments responsible for flower colors ranging from orange-red to purple and pink-magenta [7]. Indeed, flavonoids such as anthocyanins are synthesized from phenylalanine under the regulation of multiple structural genes and TFs [8,9]. Several genes have been characterized by their participation in different steps of flavonoid biosynthesis and accumulation, including those encoding phenylalanine ammonia-lyase (PAL), cinnamic acid 4-hydroxylase (C4H), 4-coumarate/CoA ligase (4CL), chalcone synthase (CHS), chalcone isomerase (CHI), flavanone 3-hydroxylase (F3H), flavonoid 3′-hydroxylase (F3′H), flavonol synthase (FLS), dihydroflavonol 4-reductase (DFR), anthocyanidin synthase (ANS), leucoanthocyanidin reductase (LAR), anthocyanidin reductase (ANR), and UDP-flavonoid glucosyltransferase (UFGT) [10,11]. These genes are mostly regulated spatiotemporally by the MYB-basic helix–loop–helix–WD40 (MBW) complex, which specifically activates flavonoid biosynthesis-related genes and, thus, affects flower color formation in plants [9,12,13,14]. In particular, R2R3-MYBs are important TFs that act as activators or repressors regulating anthocyanin biosynthesis [15]. Carotenoids are another important type of pigment that changes the flower color from yellow to red. Their accumulation mainly relies on the differential expression levels of carotenoid biosynthesis pathway (CBP) genes [16]. However, very few transcriptional regulators involved in the spatiotemporal patterns of CBP in flowers have been identified [17].

Previous studies have reported the molecular basis of color pattern formation in many plant species, including *Mimulus lewisii* [12,18], *Lilium* spp. [19,20,21], *Cattleya* [22], *Clakia gracilis* [23,24], *Phalaenopsis* spp. [25], *Paeonia suffruticosa* [26,27,28], and *Nigella orientalis* [29]. These studies revealed that the formation of flower color patterns is largely determined by the spatiotemporal regulation of the MBW complex or R2R3-MYB TFs. Thus, for example, in *P. suffruticosa*, PsMYB12 was confirmed to interact with bHLH and WD40 in a protein complex to activate *PsCHS* expression, which contributes to blotch formation [26]. Meanwhile, PsMYB30 reportedly activated *PsANS* expression to regulate blotch patterns [28]. In addition to these TFs, other TFs have also been found to be involved in blotch formation. For example, MADS-box TFs ScAG and ScAGL11 influence the appearance of the bicolor pattern in ray florets of *Senecio cruentus* [30]. Similarly, *AP3-1/4* and *AGL6-2* genes may participate in flower color pattern formation in *Cattleya* [22].

*R. persica*, which belongs to the subgenus *Hulthemia* within the Rosaceae family, is the only wild species with a dark red blotch at the base of the yellow petals and is naturally distributed in Xinjiang, China [31]. Furthermore, it is likely the main genetic resource accounting for modern rose cultivars with blotches [32]. However, to date, there is no report on the mechanism of its petal blotch formation. Here, we combined metabolome and transcriptome analyses to explore the potential mechanisms of blotch formation in *R. persica*. Our findings indicate that the differential accumulation of anthocyanins in blotch and non-blotch parts is responsible for blotch formation. Five structural genes (two *4CLs*, *DFR*, *ANS*, and *UFGT*) and 10 TFs, such as MYB, MADS, and other TFs, were identified to demonstrate similar expression patterns, displaying higher expression levels in blotch parts. These TFs may regulate structural genes to influence anthocyanin accumulation in the blotch parts. Our results contribute to elucidating the molecular mechanism of petal blotch formation in *R. persica* and provide theoretical guidance for the genetic improvement of rose cultivars.

## 2. Results

### 2.1. Metabolomic Differences among R. persica Petals

**Figure 1:**
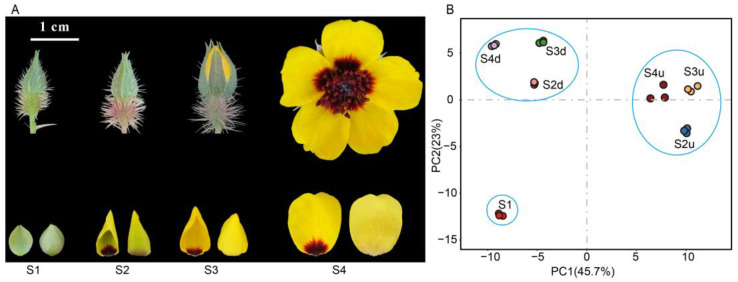
Phenotypes and PCA of flavonoids in *R. persica* flowers. (**A**): Phenotypes of *R. persica* flowers at different developmental stages. (**B**): PCA of flavonoid metabolite profiles of the petal samples. S2d, S3d, and S4d represent the blotch part of petals at three stages; meanwhile, S2u, S3u, and S4u represent the non-blotch part of the petals at three stages.

To analyze the differences in flavonoid compounds in different parts (blotch and non-blotch) of *R. persica* petals (Figure 1A), an LC-ESI-MS/MS method was used. In all, 157 flavonoids were identified, including 69 flavonols, 41 flavones, 13 flavanones, 8 flavanonols, 8 catechin derivatives, 7 anthocyanins, 4 flavone C-glycosides, 3 proanthocyanidins, 2 dihydrochalcones, 1 chalcone, and 1 isoflavone. Among these compounds, flavonols were the most abundant, including 26 quercetin and its derivatives and 23 kaempferol and its derivatives.

**Figure 2:**
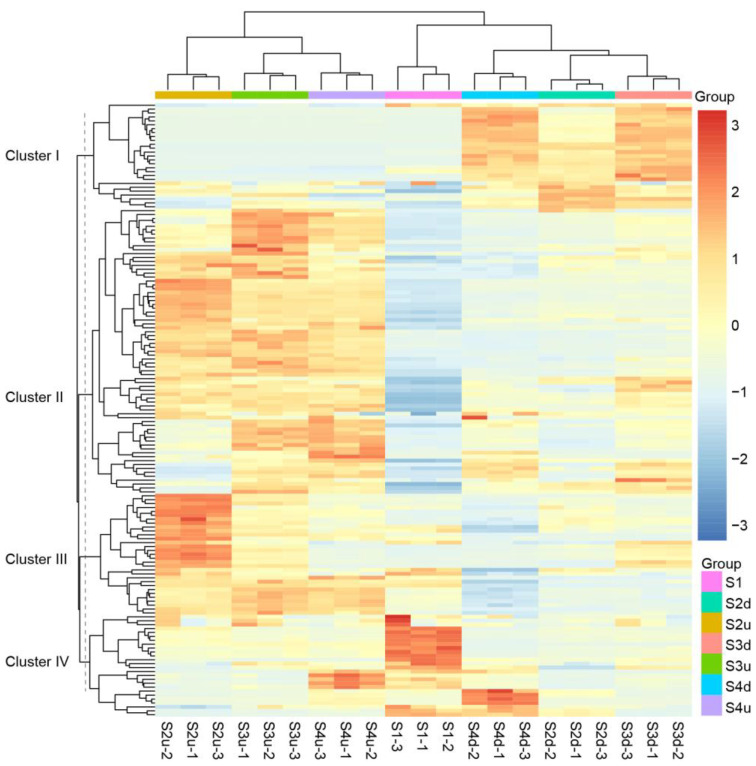
Clustering heatmap of flavonoid metabolites in 21 samples. S2d, S3d, and S4d represent the blotch part of the petals at three stages; meanwhile, S2u, S3u, and S4u represent the non-blotch part of the petals at three stages.

Principal component analysis (PCA) was performed to determine the differences in flavonoid content during the flowering stages. The first principal component (PC1) and the second one, PC2, accounted for 45.7% and 23% of the total sample variance, respectively (Figure 1B). Samples in each group were clearly distinguished. Samples at S1 were clearly separated from other groups. The samples of blotch parts from S2 to S4 were grouped together, while those of non-blotch parts were grouped together. This finding suggests that samples of the same petal parts at different stages show similar flavonoid profiles. A heatmap was drawn to show the differences in flavonoid metabolites between the blotch and non-blotch parts at different developmental stages. The result showed that samples from the same parts at different stages clustered together (Figure 2), which was consistent with that of PCA. The flavonoid metabolites in seven groups were divided into four categories. Those in Cluster I were highly accumulated in large amounts in blotches, with all anthocyanins and proanthocyanidins belonging to this cluster. Other flavonoids mainly accumulated at S1 or the non-blotch parts of the petals (Figure 2).

**Figure 3:**
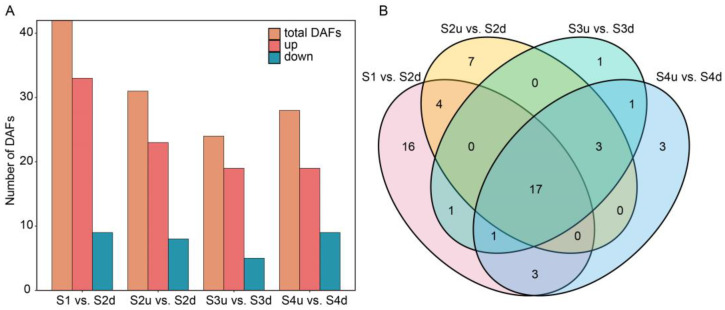
Identification of DAFs in the four pair-wise comparisons. (**A**): Number of DAFs in different comparisons. (**B**): Venn diagram of DAFs identified among comparisons. S2d, S3d, and S4d represent the blotch part of the petals at three stages; meanwhile, S2u, S3u, and S4u represent the non-blotch part of the petals at three stages.

The numbers of differentially accumulated flavonoids (DAFs) in the four comparison groups ranged from 24 to 42, with more flavonoids upregulated than downregulated (Figure 3A, Tables S1–S4). The comparison between S1 and S2d yielded the largest number of DAFs (42 flavonoids, 33 of which were upregulated and nine of which were downregulated), indicating that the accumulation of flavonoids in the petal blotch occurred before S2. These DAFs were mainly distributed in the categories of anthocyanins, flavonols, and flavones (Tables S1–S4), reflecting the differences between the blotches and non-blotch petal areas. Seventeen flavonoids, most of which were present at high levels in the blotches, were shared across all four comparisons (Figure 3B, Table S5). Seven anthocyanins including cyanidin 3-*O*-(6″-*O*-malonyl) glucoside-5-*O*-glucoside, cyanidin 3-*O*-glucoside, cyanidin 3-*O*-galactoside, cyanidin *O*-rutinoside-*O*-malonylglucoside, pelargonidin 3,5-*O*-diglucoside, pelargonidin 3-*O*-glucoside, and peonidin *O*-rutinoside-*O*-malonylglucoside, were the main shared flavonoids (41%). They were not detected or had very low abundance in the non-blotch parts of the petals. During flower development, anthocyanin abundance increased from S1 to S3 and then decreased or remained steady at S4 in the blotches (Figure S1). This trend was consistent with the color darkening of the blotch, indicating that anthocyanins contributed to a color change in the blotch formation. Moreover, other upregulated flavonoids may act only as background colors.

In the comparisons of S3u vs. S3d and S4u vs. S4d, a total of 11 DAFs were identified to be downregulated in the blotch parts, including 2 flavanones, 4 flavonols and 5 flavones. Among the flavonols, kaempferol 3-*O*-(6″-p-coumaroyl) galactoside, quercetin 3-*O*-(2″-cinnamoyl) glucoside, and quercetin 3-*O*-(6″-p-coumaroyl) glucoside displayed higher accumulation in the non-blotch parts at S3 and S4 (Figure S2), indicating that they might possibly be related to the yellow non-blotch of petals. On the other hand, the relative content of flavanones either decreased or the difference was not significant in non-blotch samples from S2 to S4 and 5 flavones were highest at S1.

### 2.2. Differential Accumulation of Carotenoids in the Petals of R. persica

**Figure 4:**
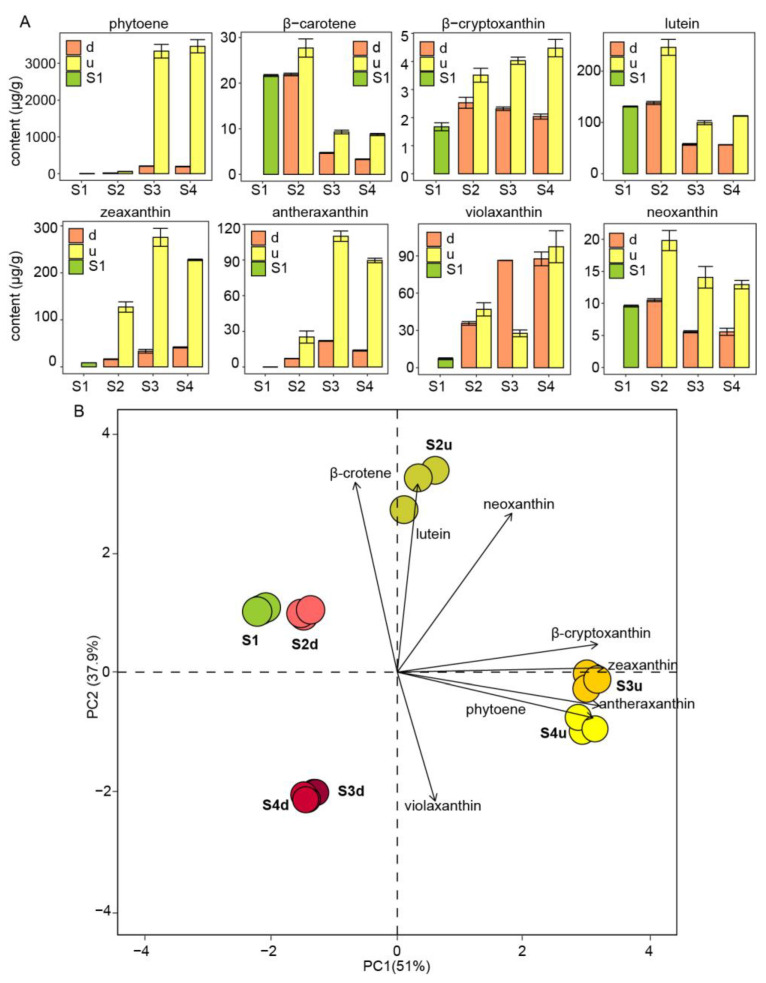
Major carotenoids identified and PCA of their levels in petals during different flower developmental stages of *R. persica.* (**A**): Carotenoid contents in petals at four developmental stages. (**B**): PCA of carotenoids in petals at four stages. d and u represent blotch and non-blotch parts of petals, respectively; S2d, S3d, and S4d represent the blotch part of petals at three stages; meanwhile, S2u, S3u, and S4u represent the non-blotch part of petals at three stages.

To elucidate the role of carotenoids in petal color formation, a targeted metabolomic experiment was conducted through LC-MS/MS. Fourteen carotenoid types were identified in the petals of *R. persica* during flower development, six of which were present at low levels (Table S6). Carotenoids accumulated to a much higher in the non-blotch parts. Furthermore, the most abundant carotenoid was phytoene, reaching levels higher than 3000 μg·g^−1^ in non-blotches at S3 and S4 (Figure 4A). Five xanthophylls (zeaxanthin, lutein, antheraxanthin, violaxanthin, and neoxanthin) were the major downstream products of the carotenoid biosynthesis pathway. Among them, lutein, zeaxanthin, and antheraxanthin were three abundant carotenoids, accounting for more than 80% of all xanthophylls. In the non-blotch parts, the concentrations of zeaxanthin and antheraxanthin increased from S2 to S4 and peaked at S3, causing the upper part of the petals to turn completely yellow. Moreover, the lutein content was highest in the non-blotch part at S2, decreasing at S3 and S4. The different accumulation patterns of these carotenoids contributed to the coloration of green-to-yellow petals during flower development. Consequently, the accumulation of zeaxanthin and antheraxanthin resulted in the yellow color of the non-blotch parts of petals in *R. persica*.

A PCA of the eight major carotenoids at four stages was also performed. PC1 and PC2 explained 51% and 37.9% of the total variance, respectively (Figure 4B). No characteristic carotenoids were found at S1 or in the petal blotches at any stage. The non-blotches at S2, located in the first quadrant, were characterized by higher lutein levels. Meanwhile, the non-blotches at S3 and S4, located in the fourth quadrant, were characterized by high phytoene, zeaxanthin, and antheraxanthin contents. Different parts of the petals at the four stages could be distinguished based on their carotenoid profiles, demonstrating that the carotenoid profiles differed significantly among the samples. The results of PCA proved that the yellow color of non-blotch parts at S3 and S4 was related to the accumulation of zeaxanthin and antheraxanthin.

### 2.3. Transcriptomic Analysis of R. persica

RNA-seq was performed on samples of petals with blotch and non-blotch parts at the four stages during flowering. After removing low-quality sequences, each library had 39,898,184–57,227,586 clean reads. The Q30 and contents of GC were 92.85–94.68% and 46.68–48.41%, respectively. The mapped ratio of all clean reads mapping the *R. persica* genome was higher than 91% in each sample (Table S7). The PCA results showed that PC1 and PC2 explained a total variation of 81.6% (Figure S3A). Pearson’s correlation coefficient (PCC) revealed that petal blotch and non-blotch samples at the same stage exhibited a significant correlation. Furthermore, it revealed that intra-group uniformity was strong (Figure S3B). These results confirmed the high quality of the transcriptome data.

### 2.4. Differentially Expressed Gene (DEG) Analysis of Petal Blotch and Non-Blotch Parts at Four Stages

**Figure 5:**
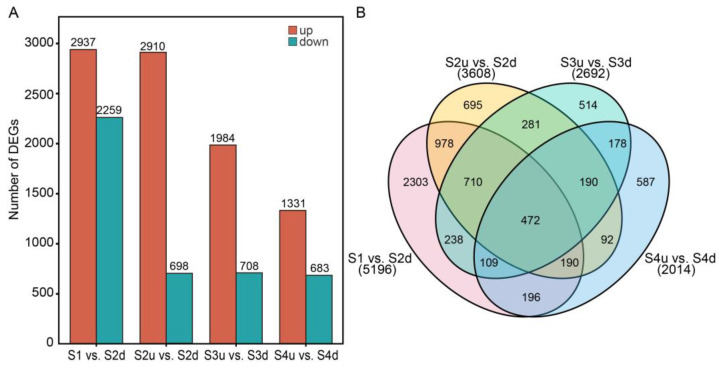
Identification of DEGs in the four pair-wise comparisons. (**A**): Numbers of up- and downregulated DEGs in different pair-wise comparisons. (**B**): Venn diagram of DEGs identified among comparisons. S2d, S3d, and S4d represent the blotch part of the petals at three stages; meanwhile, S2u, S3u, and S4u represent the non-blotch part of the petals at three stages.

Differential expression analyses were conducted between four comparison groups. In all, 7733 DEGs were identified in the comparisons of S1 vs. S2d, S2u vs. S2d, S3u vs. S3d, and S4u vs. S4d. The number of DEGs ranged from 2014 to 5196 in each comparison (Figure 5A). In the four comparisons, the number of upregulated genes was much higher than the downregulated genes, and the highest number was found in the comparison of S1 vs. S2d, which was similar to the difference in DAFs. DEGs may play a crucial role in regulating petal coloration. There were 472 common DEGs detected among the four comparisons (Figure 5B). According to the KEGG functional enrichment analysis, carotenoid biosynthesis and the phenylpropanoid pathway were enriched in all comparisons. In the S2u vs. S2d, S3u vs. S3d, and S4u vs. S4d comparison groups, flavonoid biosynthesis pathways were consistently enriched (Figure S4). We screened the DEGs related to anthocyanin biosynthesis, including *PAL*, *4CL*, *CHS*, *F3H*, *DFR*, *ANS*, *LAR*, *ANR*, and *UFGT*. All these genes were annotated as members of flavonoid and anthocyanin biosynthesis pathways. 

### 2.5. Expression Profiles of Genes Related to Anthocyanin Biosynthesis Involved in Blotch Formation

**Figure 6:**
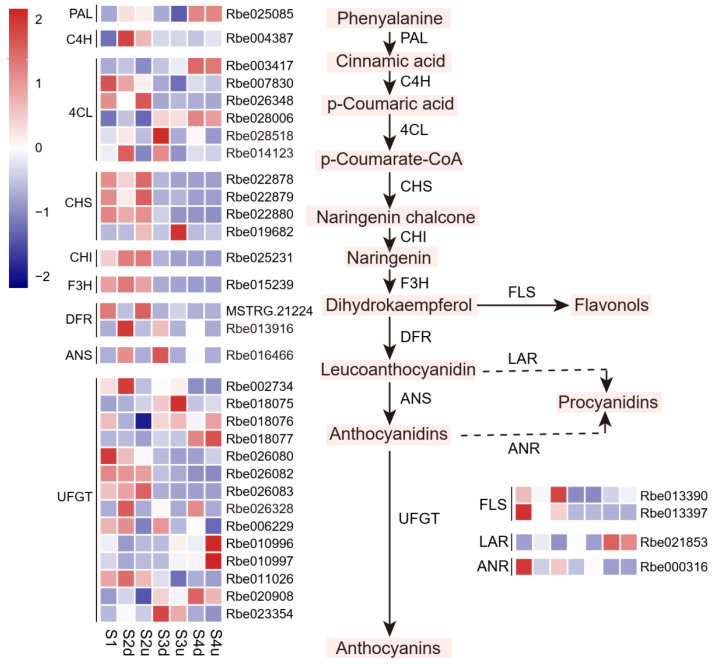
Differential expression profiles of anthocyanin biosynthesis pathway-related genes in different petal parts during *R. persica* flower development in *R. persica*. S2d, S3d, and S4d represent the blotch part of the petals at three stages; meanwhile, S2u, S3u, and S4u represent the non-blotch part of the petals at three stages.

To further investigate the mechanism of anthocyanin accumulation in the petal blotch, the expression levels of genes in the anthocyanin pathway were analyzed, and a heatmap of DEGs was constructed. In this case, 35 genes were identified as DEGs (Figure 6). The PCC between the abundance of the seven anthocyanins and the expression levels of DEGs were calculated. The results showed that the expression of five genes, namely *4CL* (Rbe014123 and Rbe028518), *DFR* (Rbe013916), *ANS* (Rbe016466), and *UFGT* (Rbe026328), were positively correlated with anthocyanin content in the samples (r > 0.75, *p* < 0.05, Table S8). These genes were specifically expressed in the blotches, suggesting that they might have an important effect on the accumulation of anthocyanins in such parts.

### 2.6. Identification of TFs Involved in Blotch Formation

**Figure 7:**
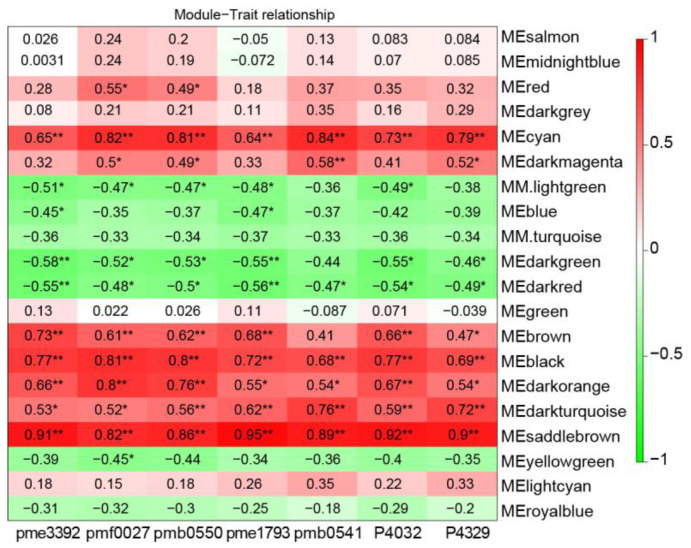
Module–anthocyanin relationship analysis. The value inside the box represents the PCC value between the module with anthocyanin. * *p* < 0.05 and ** *p* < 0.01 (Student’s *t*-test).

**Figure 8:**
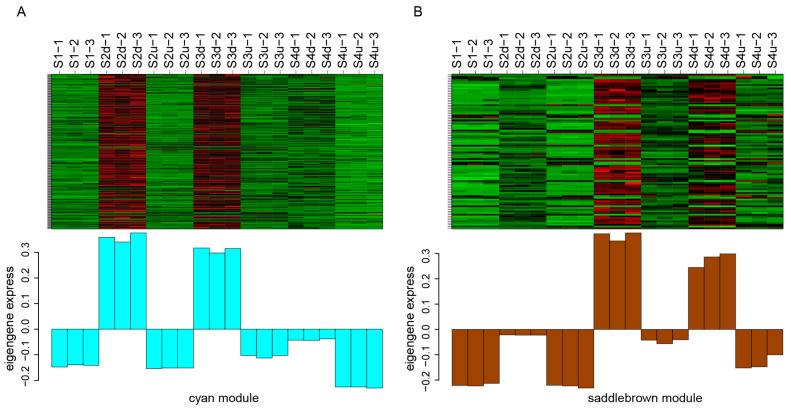
The expression level of DEGs in the cyan and saddlebrown modules (**A**): The expression level of DEGs in the cyan module. (**B**): The expression level of DEGs in the saddlebrown module. The red color represents genes are up-regulated, and the green color represents genes are down-regulated. S2d, S3d, and S4d represent the blotch part of the petals at three stages; meanwhile, S2u, S3u, and S4u represent the non-blotch part of the petals at three stages.

Weighted gene co-expression network analysis was performed to characterize the co-regulatory factors involved in petal blotch formation. After filtering, 7167 DEGs (FPKM ≥ 1) were merged into 20 distinct modules (Figure S5). The correlation coefficients between the modules and the anthocyanins were then calculated. The results showed that the two modules (cyan and saddlebrown) were significantly and positively correlated with more than three anthocyanins (r > 0.8, *p* < 0.05, Figure 7). Notably, genes in the cyan and saddlebrown modules showed higher expression levels in the blotch than those in the non-blotch part of the petals (Figure 8A,B). The saddlebrown module comprised 92 genes, none of which were involved in the flavonoid pathway, as well as five TFs. The cyan module contained 269 genes, including 4 genes (2 *4CLs*, *DFR*, and *ANS*) that were identified to be closely related to anthocyanin accumulation. Additionally, KEGG enrichment analysis revealed that genes in the cyan module were enriched in flavonoid biosynthesis pathways (Figure S5). Therefore, this module was further analyzed, and it was found to contain 22 TFs, including members of the MYB, NAC, MADS, and other families (Table S9). Among these TFs, 10 showed comparatively high positive correlation coefficients with anthocyanins (r > 0.8, *p* < 0.05; Table S10), indicating that they may be essential to anthocyanin synthesis.

### 2.7. qRT-PCR Validation of DEGs

**Figure 9:**
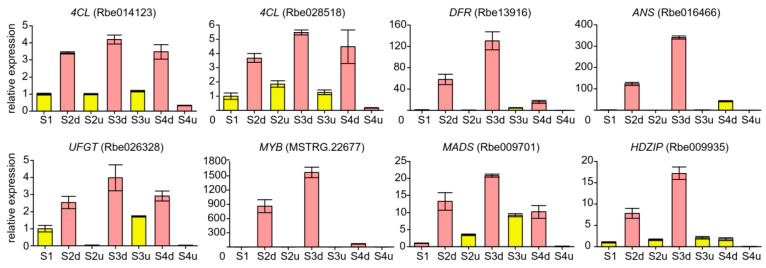
Relative expression levels of candidate genes from qRT-PCR analysis. Three independent biological experiments were performed. S2d, S3d, and S4d represent the blotch part of the petals at three stages; meanwhile, S2u, S3u, and S4u represent the non-blotch part of the petals at three stages.

To validate the transcriptome data, five candidate structural genes and three TFs were selected to analyze the corresponding relative spatiotemporal expression by means of qRT-PCR analysis. According to the qRT-PCR results, the structural genes (two *4CLs*, *DFR*, *ANS*, and *UFGT*) were expressed at higher levels in the blotch parts than in the non-blotch parts of petals; in particular, *DFR* and *ANS* were highly expressed in the blotch part of petals, with their relative expression levels increasing and then decreasing, and peaking at S3 (Figure 9). These five genes are supposed to be the key structural genes responsible for the anthocyanin accumulation in the blotch parts of petals. Additionally, we verified the expression levels of *MYB*, *MADS*, and *HDZIP*, which showed the same trends as that of structural genes. In particular, the relative expression level of MYB (MSTRG. 22677) was significantly high in the blotch part at S2 and S3 (Figure 9). Furthermore, qRT-PCR analysis showed that structural genes and TFs related to anthocyanin synthesis showed a similar pattern to that of the anthocyanin content, indicating that the accumulation of anthocyanins in the blotch parts might be attributed to structural genes (*4CL*, *DFR*, *ANS*, and *UFGT*) and TFs. Additionally, the results also proved that the transcriptome data were reliable.

## 3. Discussion

The formation of flower color patterns is usually a consequence of the accumulation of pigment differences in different areas of the petals [3]. Flavonoids, especially anthocyanins, are an important class of secondary plant metabolites [33] that are reportedly closely related to flower coloration [7] and whose differential accumulation determines petal blotch formation [34]. For example, petal blotches in Xibei tree peonies were directly caused by differential flavonoid accumulation in different parts of the petals [35]. In *Phalaenopsis*, the higher accumulation of anthocyanins at different parts of the petals resulted in a petal blotching color pattern [36]. Consistently, in this study, 157 flavonoids were identified in the petals of *R. persica* at four flower developmental stages. Seven anthocyanins, including cyanidin 3-*O*-(6″-*O*-malonyl) glucoside 5-*O*-glucoside, cyanidin-3-*O*-glucoside, cyanidin 3-*O*-galactoside, cyanidin *O*-rutinoside-*O*-malonylglucoside, pelargonidin 3-*O*-glucoside, pelargonidin 3,5-*O*-diglucoside, and peonidin *O*-rutinoside-*O*-malonylglucoside, were the main upregulated DAFs across four comparisons. Moreover, the amounts of these anthocyanins in blotches were significantly higher and showed an increasing trend during flower development, decreasing or remaining stable thereafter. The accumulation pattern of anthocyanins was consistent with the blotch pigmentation of petals during flower development. Thus, we speculated that these seven anthocyanins participated in blotch formation and color darkening. This finding was different from the finding that only cyanidin 3,5-*O*-diglucoside contributed to the blotch pigmentation of ‘Sunset Babylon Eyes’ [6]. Other flavonoids, chalcones, flavonols, and flavones mainly contributed to the yellow flower coloration. In our study, three flavonols were identified to display high accumulation in the non-blotch parts, indicating that they might be related to yellow coloration. It was consistent with the findings of a similar study on ‘Sunset Babylon Eyes’ that flavonols acted as background colors [6]. 

Carotenoids are the most abundant pigments that contribute to the yellow flowers. In this study, 14 carotenoids were detected in the petals of *R. persica*. The accumulation of different carotenoids was confirmed as the cause of coloration in the non-blotch parts of *R. persica* petals. During flower development in *R. persica*, zeaxanthin, and antheraxanthin were the predominant carotenoids at S3 and S4, causing the yellow coloration of non-blotch parts. Furthermore, the colorless phytoene was the most abundant carotenoid at the blooming stage, providing sufficient precursors for downstream carotenoid synthesis. Similar results were previously obtained for *Capsicum annuum* [37]. Flavonols were always colorless or pale yellow. Considering the differential accumulation of carotenoids in different parts of the petals, we speculate that carotenoids predominantly contribute to the yellow coloration of petals in *R. persica*. Whether there is an interactive effect on the coloration between flavonols and carotenoids remains to be determined in further study.

The spatiotemporal differential expression of structural genes that control pigment synthesis allegedly plays a critical role in petal blotch formation [38]. Considering the difference in anthocyanin abundance in different parts of *R. persica* petals, five DEGs (namely two *4CLs* and one each of *DFR*, *ANS*, and *UFGT*) related to anthocyanin synthesis were identified in this study. As upstream genes in flavonoid/anthocyanin biosynthesis, *4CL* enzymes are crucial for anthocyanin accumulation through a series of chemical reactions [39]. For example, in *Camellia japonica*, *4CL* was significantly upregulated in the petals of darkened color varieties compared to the corresponding expression level in the white variety [40]. Similarly, *4CL* expression was upregulated in dark-gray, purple leaves compared to reddish-purple leaves in purple tea [41]. In this study, the higher anthocyanin content in the blotches was likely related to the upregulation of *4CL* expression levels after considering the expression of the two *4CL* genes (Rbe014123 and Rbe028518).

The key *DFR*, *ANS*, and *UFGT* genes are downstream of the anthocyanin biosynthesis-related genes. These three genes of vital importance in blotch formation have been previously reported for different species. Additionally, four structural genes, *ScCHS2*, *ScF3H1*, *ScDFR3*, and *ScANS*, were inhibited in the colorless regions of the bicolor cultivars of *S. cruentus* [30]. Similarly, the differentially expressed anthocyanin structural genes of *PsCHS, PsF3′H*, *PsDFR*, and *PsANS* were expressed at a significantly higher level in the spot than the non-spot areas of the petals of *P. suffruticosa* [42]; PhUGT78A22 can catalyze the conversion of cyanidin-3-*O*-glucoside and peonidin-3-*O*-glucoside to cyanidin-3,5-*O*-glucoside and peonidin-3,5-*O*-glucoside during blotch formation [43]. In this study, *DFR* (Rbe013916), *ANS* (Rbe016466), and *UFGT* (Rbe026328) were specifically highly expressed in the blotch parts of *R. persica* petals. Their expression patterns were consistent with the expression of *PsDFR*, *PsANS*, and PhUGT78A22 in peonies [42,43,44]. Moreover, the abundance of anthocyanins showed a similar trend, which could be attributed to the high expression of these aforementioned genes in the blotch parts of petals. Additionally, the expression levels of these five genes increased rapidly from S1 to S2 (Figure 9). Anthocyanin accumulation also increased rapidly as the blotches appeared from S1 to S2. Furthermore, the expression pattern of these genes was consistent with the accumulation of anthocyanins in the petals during flower development. Therefore, we speculated that the differential expression of these five genes significantly contributes to blotch formation in *R. persica* petals.

Transcription factors are essential for regulating structural genes and color pattern formation. Particularly, MYB TFs are the key determinants of anthocyanin biosynthesis, and their temporal and spatial expression patterns influence the patterns of anthocyanin distribution [3,45,46]. In turn, FaMYB5’s interaction with FaBBX24 promoted the expression of *F3′H*, *4CL-2*, and other genes to increase anthocyanin and proanthocyanidin in strawberry fruit [47]. Meanwhile, CgMYB1 activated *CgDFR2* and *CgANS* to produce petal spots in *C. gracilis* [24]. Similarly, LhMYB18 interacted with LhbHLH2 to activate the promoter of *DFR*, which was responsible for large spot formation in an Asiatic hybrid lily cultivar [19], while HaMYB1 modulated *UFGT* to promote anthocyanin accumulation in *Helianthus annuus* [48]. Recently, *RcMYB1* [49], *RhMYB114a* [50], *RhMYB3b* [50], and *Rosa1* [51] were shown to play roles in anthocyanin synthesis in roses. In this study, three MYBs and one MYB-related TF were highly associated with anthocyanins in *R. persica*. They were significantly upregulated in the blotch parts, and their expression trends were consistent with those of the characterized structural genes. Other developmental regulatory genes and environmental factors also affect anthocyanin synthesis [52]. For instance, MADS-box TFs *OAGL6-1* and *OAGL6-2* were involved in anthocyanin pigmentation and red spot formation in orchids, respectively [53]. Similarly, two *R2R3-MYBs* (*DPL* and *PHZ*) regulate the pigmentation pattern of flowers, and transcripts for *PHZ* are induced by high light in petunias [54]. In turn, light-responsive RhHY5 positively activates the expression of *RhMYB114a* and represses the expression of *RhMYB3b* to regulate anthocyanin biosynthesis in rose petals [50]. The overexpression of a C2H2-type zinc finger protein gene *MdZAT5* in apple calli and *Arabidopsis* enhances anthocyanin accumulation [55]. Consistently, we found that some differentially expressed TFs, including MADS-box, C2H2, HD-ZIP, WD40, G2-like, and TALE, were positively related to anthocyanin and structural genes. These TFs may function in regulating anthocyanin accumulation in the petals of *R. persica*. Although we screened several candidate TFs possibly involved in anthocyanin biosynthesis, further research is required to confirm their functions. Therefore, future experiments will focus on gene overexpression or knockdown experiments to elucidate the role of these candidate genes in anthocyanin accumulation and blotch formation in the petals of *R. persica*.

## 4. Materials and Methods

### 4.1. Plant Materials

The flower petals of *R. persica* were collected in Changji Hui Autonomous Prefecture, Xinjiang Uygur Autonomous Region of China (N 44°10′, E 86°46′, H 570). Flower development was divided into four stages (Figure 1A): the green flower bud stage, at which the length of the bud was 0.94 ± 0.18 cm (S1) and petals were without a blotch; the yellow-green flower bud stage, at which the length of the bud was 1.12 ± 0.19 cm, and blotch appeared at the base of petals (S2); the semi-open stage, at which the length of the bud was 1.31 ± 0.59 cm, and the non-blotch part changed to yellow (S3); and the full bloom stage, at which the diameter of the flower was 3.15 ± 0.74 cm (S4). Petals were collected at each of these four stages, and the petal blotches and non-blotches were separated and sampled at stages S2–S4. Three biological replicates were included for pigment analysis and RNA extractions. For each replicate, 15 uniform flowers were collected at S1, S3, and S4, while 30 uniform flowers were collected at S2. All samples were collected in May 2020 between 8:00 a.m. and 11:00 a.m. on sunny days and stored in a refrigerator at −80 °C until used.

### 4.2. Metabolomic Analysis of Flavonoids and Carotenoids

For flavonoid metabolomics, the freeze-dried flower petals of *R. persica* were ground into a powder. Then, 0.1 g of powder was extracted with 1.0 mL of a 70% methanol aqueous solution overnight at 4 °C. After centrifugation at 10,000× *g* for 10 min, the supernatants were filtrated through a 0.22 μm microporous membrane for further analysis. Sample extracts were analyzed using an LC-MS/MS system [37]. Flavonoids were identified using the MWDB database (Metware Biotechnology Co., Ltd., Wuhan, China) and public databases. DAFs were identified based on the criteria of a VIP (variable importance in the project) value ≥ 1, |Log_2_FC (fold change)| ≥ 1, and *p*-value < 0.05. 

For carotenoid metabolomics, 0.05 g of freeze-dried flower petal powder was extracted with a 1 mL solution composed of n-hexane/acetone/ethanol (2:1:1, *v*:*v*:*v*) with 0.01% BHT. An internal standard was added for quantitative analysis. The mixture was then vortexed for 20 min at room temperature. The supernatants were harvested after centrifugation of 12,000 rpm and 4 °C for 5 min. The residue was subsequently re-extracted by repeating the above steps, and the supernatants were combined and dried. The dried residues were dissolved with 100 μL of a mixture of methanol and methyl tert-butyl ether (MTBE) (1:1). The solution was vortexed for 30 s and centrifuged at 12,000 rpm and 4 °C for 5 min. Finally, the sample was filtered through a 0.22 μm membrane and stored in brown sample bottles for further analysis. The characterization of the sample extracts was conducted using an LC-APCI-MS/MS system, as described previously [56]. The carotenoid contents were quantified using calibration curves based on 18 standards. Three replicates were included for each assay.

### 4.3. Transcriptome Sequencing and Analyses

A total of 21 libraries were constructed and sequenced by Gene Denovo Biotechnology (Guangzhou, China) using the Illumina HiSeq2500 platform. Each sample was performed in triplicate. After removing the adaptor and low-quality sequences, the cleaned reads were mapped to the *R. persica* genome (unpublished) using the HISAT2.2.4 software [57]. The parameter ‘-rna-strandness RF’ was set, and the other parameters were set as a default. Gene expression levels were quantified by fragments per kilobase of the exon model per million read (FPKM) values calculated by RSEM 1.2.19 [58]. DEGs in the samples were identified using the DESeq2 package [59]. Genes with the |log_2_FC| ≥ 1 and false discovery rate (FDR) < 0.05 were characterized as differentially expressed. Subsequently, the DEGs were subjected to enrichment analysis by the Kyoto Encyclopedia of Genes and Genomes (KEGG) database.

### 4.4. Weighted Gene Co-Expression Network Analysis

The DEGs from four comparison groups (S1 vs. S2d, S2u vs. S2d, S3u vs. S3d, and S4u vs. S4d) were used to identify the modules involved in the petal blotch using the WGCNA package in RStudio 4.2.1. The soft-threshold power of the correlation analysis was set to eight, at which point the correlation coefficient between the genes was found to be 0.8, and the mean connectivity was stable. The minimum number of genes in each module size was 30. A KEGG pathway enrichment analysis was performed on the genes in each module to clarify their biological functions. PCC was calculated between seven anthocyanins and eigengenes of each module. The module with r > 0.8 and *p* < 0.05 was identified to be involved in anthocyanin biosynthesis.

### 4.5. Real-Time Quantitative Reverse Transcription PCR (qRT-PCR) Analysis

The extraction of the total RNA from different parts of *R. persica* petal samples collected at four developmental stages was conducted using the TRIzol reagent kit (Invitrogen, Carlsbad, CA, USA). cDNA was synthesized from the total RNA of *R. persica* flowers using the PrimeScript^TM^ RT reagent kit and gDNA Eraser (TaKaRa, Kusatsu City, Japan). Primers for qRT-PCR were designed using the PrimerQuest Tool (https://sg.idtdna.com/PrimerQuest/Home/Index, accessed on 25 April 2021). All the primers used are listed in Table S11. *RhGAPDH* was used as a reference gene to normalize the gene expression. Reactions were conducted on a LightCycler 480 II System (Roche, Basel, Switzerland) using the NovoStart^®^ SYBR High-Sensitivity qPCR SuperMix (Novoprotein, Suzhou, China). The qRT-PCR procedure was as follows: 95 °C for 1 min, 95 °C for 20 s, and 60 °C for 45 s over 40 cycles. The relative expression levels of each gene were analyzed by the 2^−ΔΔCt^ method [60]. Three biological replicates were analyzed for each sample.

### 4.6. Statistical Analysis

Data were analyzed using SPSS Statistics 20.0 software (IBM, Chicago, IL, USA). PCA and heatmaps were generated using the factoextra and pheatmap packages in RStudio software, respectively.

## 5. Conclusions

In this study, integrated metabolome and transcriptome analyses were first performed to identify the key pigments and genes responsible for the petal blotch formation of *R. persica*. In all, 157 flavonoids were detected in different parts of the petals during flower development. The blotch formation and color darkening were mainly caused by the abundant accumulation of anthocyanins. In contrast, carotenoids were found to be predominantly responsible for the formation of the yellow color in the non-blotch parts. Five structural genes, namely two *4CLs* (Rbe014123 and Rbe028518), *DFR* (Rbe013916), *ANS* (Rbe016466), and *UFGT* (Rbe026328), as well as ten differentially expressed TFs, were screened as candidate regulators contributing to petal blotch formation. However, the complex regulatory mechanisms underlying the differential accumulation of pigments warrant further research. The functional validation of candidate genes is beneficial to fully understand the mechanism of blotch formation. Overall, our findings provide an important foundation for understanding the formation of petals in *R. persica*, which, in turn, can be used for selective breeding or genetic engineering for novel rose cultivars with floral blotch.

## Data Availability

The raw data in transcriptome for this study were submitted to the National Center for Biotechnology Information (NCBI) under project number PRJNA1068021.

## Supplementary Materials

The following supporting information can be downloaded at: https://www.mdpi.com/article/10.3390/ijms25074030/s1.
