# Supplementary material for: Integrative Metabolomic and Transcriptomic Analyses Reveal the Mechanism of Petal Blotch Formation in Rosa persica
Source: Int J Mol Sci. 2024 Apr 4;25(7):4030. doi: 10.3390/ijms25074030 (PMC11012444; doi:10.3390/ijms25074030)
Supplement: Supplementary file 1 [file ijms-25-04030-s001.zip › ijms-2906296-supplementary.pdf]

Supplementary Figures

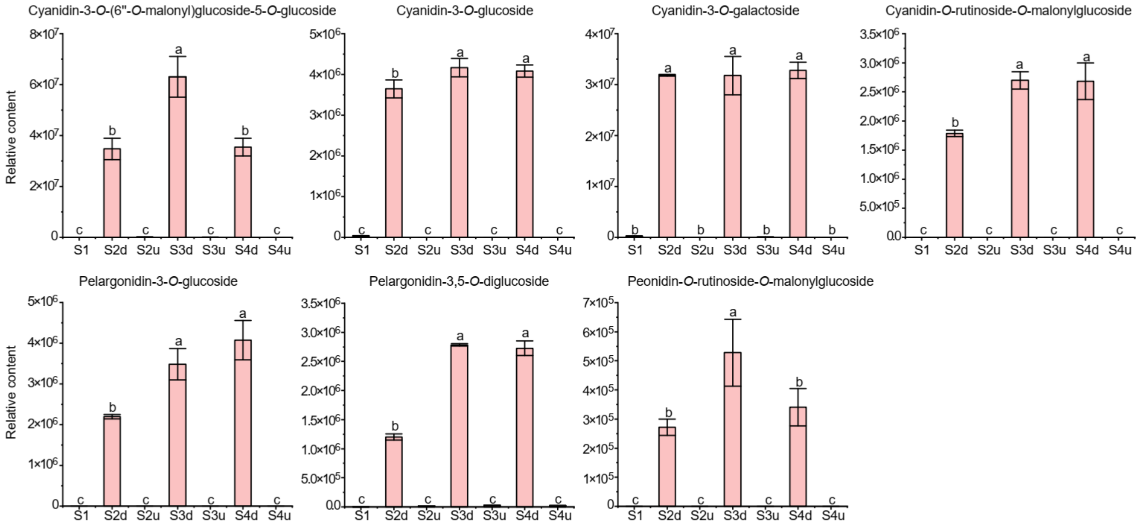

Figure S1. The relative content of seven anthocyanins in the blotch and non-blotch petal parts of petals in *R. persica.*

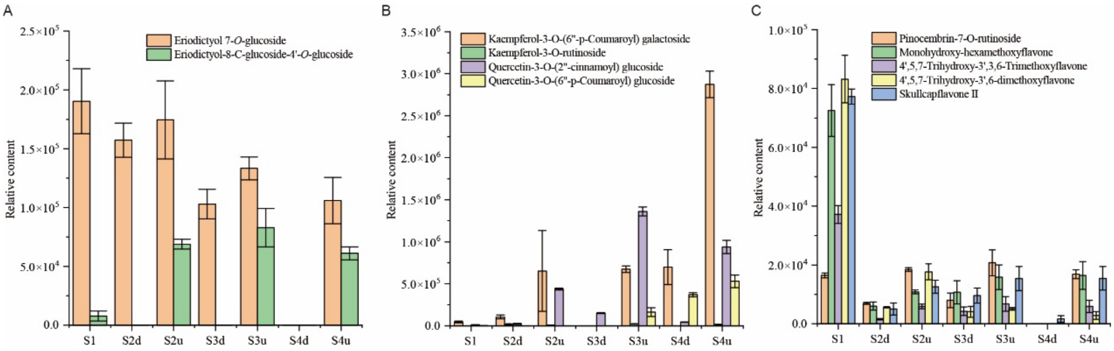

Figure S2. The relative content of 11 DAFs in the comparisons of S3u vs. S3d and S4u vs. S4d in *R. persica.* **(A):** The relative content of flavanones. **(B):** The relative content of flavonols. **(C):** The relative content of flavones.

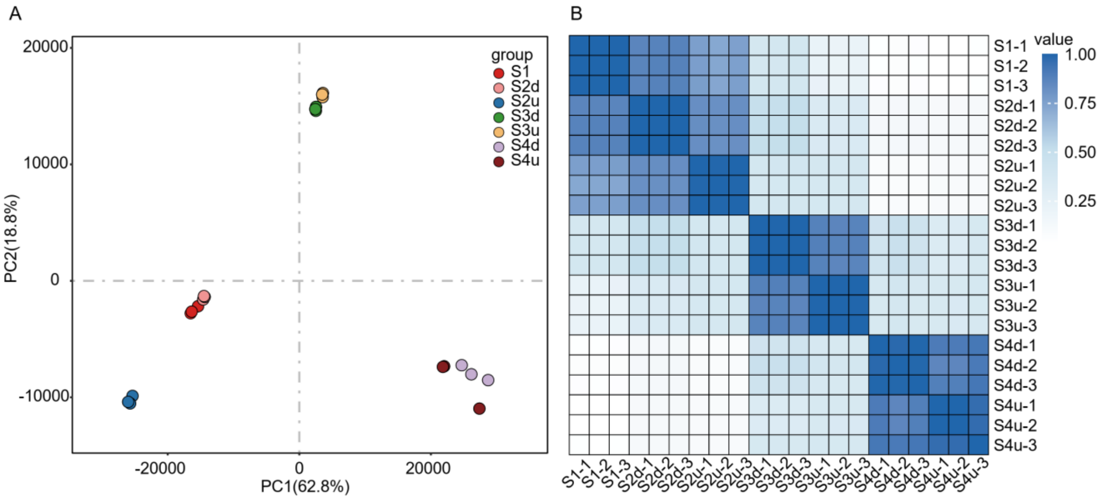

Figure S3. RNA-seq analysis of petal blotch and non-blotch samples across 21 sequencing libraries. **(A):** Principal component analysis of gene expression profile across samples. **(B):** Pearson correlation analysis of RNA samples across 21 sequencing libraries.

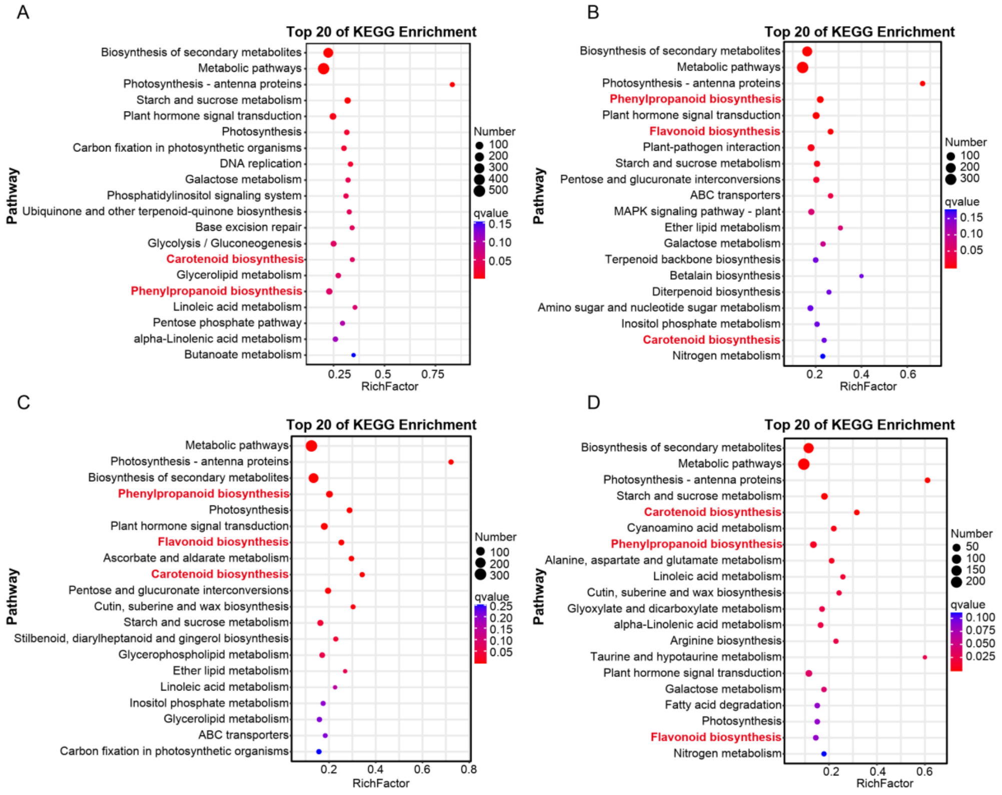

Figure S4. KEGG enrichment analysis of DEGs identified in four comparisons. **(A):** S1 vs. S2d. **(B):** S2u vs. S2d. **(C):** S3u vs. S3d. **(D):** S4u vs. S4d.

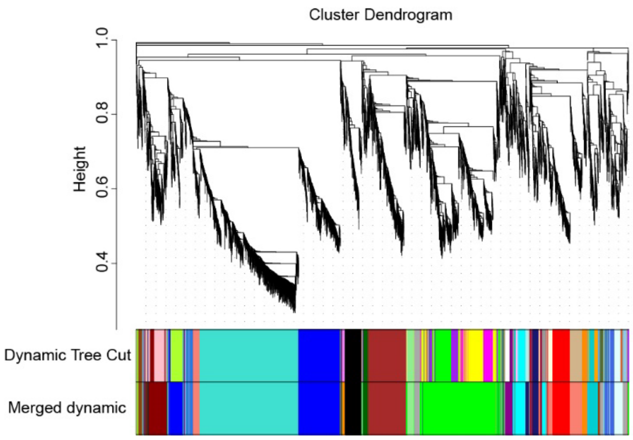

Figure S5. Hierarchical clustering dendrogram of 20 gene expression modules

Supplementary Tables

Table S1. Differentially accumulated flavonoids between S1 and S2d.

Index	Compounds	Class	S1-1	S1-2	S1-3	S2d-1	S2d-2	S2d-3	Log_2FC	VIP	Type
pme3392	Pelargonidin-3-O-glucoside	Anthocyanins	9	9	9	2237700	2140100	2210200	17.897	2.178	up
P4032	Cyanidin-O-rutinoside-O-malonylglucoside	Anthocyanins	9	9	9	1728000	1793100	1842400	17.600	2.160	up
P4329	Peonidin-O-rutinoside-O-malonylglucoside	Anthocyanins	9	9	9	241770	297100	275640	14.881	1.985	up
pmb0541	Cyanidin-3-O-(6''-O-malonyl) glucoside-5-O-glucoside	Anthocyanins	36216	18013	16599	30223000	38450000	35650000	10.524	1.675	up
pme1793	Pelargonidin-3,5-O-diglucoside	Anthocyanins	1241	1821	858	1257200	1201200	1146500	9.845	1.619	up
pmf0027	Cyanidin-3-O-galactoside	Anthocyanins	279570	233230	239970	31798000	31682000	32052000	6.988	1.361	up
pmb0550	Cyanidin-3-O-glucoside	Anthocyanins	32345	38713	30723	3424500	3863700	3658200	6.749	1.338	up
HJN041	Epicatechin glucoside	Catechin derivatives	9	9	9	155130	168800	158920	14.126	1.935	up
pmb0628	Eriodictyol-8-C-glucoside-4'-O-glucoside	Flavanone	3061	11560	8864	9	9	9	-9.765	1.586	down
mws0463	Hesperetin	Flavanone	9	9	9	6398	5933	7028	9.486	1.585	up
mws1174	3-O-Acetylpinobanksin	Flavanonol	16647	18083	11190	9	9	9	-10.732	1.683	down
pmb2979	Hesperetin-7-O-(6''-malonyl) glucoside	Flavanonol	2001	3184	4424	116800	128070	118960	5.243	1.182	up
Lmjp005224	4',5,7-Trihydroxy-3',3,6-Trimethoxyflavone	Flavone	33739	38322	39495	1497	1844	1358	-4.569	1.100	down
Zmhp004269	Skullcapflavone II	Flavone	78977	74258	78470	4051	7358	3685	-3.940	1.025	down
pmp000004	4',5,7-Trihydroxy-3',6-dimethoxyflavone	Flavone	73934	89037	86651	5441	5809	5634	-3.886	1.014	down
pmp000786	3',5-Dihydroxy-4',6,7-trimethoxyflavone	Flavone	16173	21006	18736	1846	593	1564	-3.804	1.007	down
mws1661	Diosmin	Flavone	9	9	9	337560	306580	328650	15.137	2.003	up
mws0920	Tricetin	Flavone	9	9	9	116900	148520	129860	13.838	1.914	up
pmb0592	Chrysoeriol-7-O-rutinoside-5-O-glucoside	Flavone	9	9	9	133650	109410	119860	13.714	1.906	up
Hmjn004446	Luteolin-7-O-(6''-caffeoyl) rhamnoside	Flavone	133310	119150	121550	2219700	2100500	2158200	4.114	1.044	up
Hmhp005228	Norwogonin	Flavone	33739	30732	27063	422030	485330	463680	3.905	1.017	up
mws1608	Luteolin-6-C-glucoside	Flavone C-glycosides	18263	20289	18823	9	9	9	-11.053	1.711	down

pmb0672	Apigenin-6-C-glucoside-7-O-(6"-feruloyl) glucoside	Flavone C-glycosides	9	9	9	111230	110400	119520	13.625	1.900	up
mws0988	Rhamnetin	Flavonol	7744	8808	8285	9	9	9	-9.845	1.615	down
Lmdp004267	Quercetin-3-O-(6"-p-Coumaroyl) glucoside	Flavonol	2480	2475	331	9	9	9	-7.613	1.351	down
Lmjp002867	Kaempferol-3-O-neohesperidoside	Flavonol	9	9	9	9904800	9401300	9283600	20.014	2.303	up
Lmpp003268	Kaempferol-3-O-rutinoside-7-O-glucoside	Flavonol	9	9	9	5416700	4473000	4864850	19.060	2.247	up
Lmyn001269	Kaempferol-3-O-sophoroside	Flavonol	9	9	9	673810	630110	629850	16.128	2.067	up
pmp001312	6-Hydroxykaempferol-3,7,6-O-triglycoside	Flavonol	9	9	9	52964	51099	53020	12.506	1.820	up
pmn001644	Quercetin-3-O-(2"-acetyl) glucuronide	Flavonol	9	9	9	46884	44419	46230	12.315	1.806	up
pmb0709	Quercetin-7-O-(6"-malonyl) glucosyl-5-O-glucoside	Flavonol	9	9	9	28152	29118	27850	11.622	1.755	up
Lmsn002815	Kaempferol-3-O-rutinoside	Flavonol	9	9	9	14383	23543	17654	11.007	1.705	up
pmn001640	Myricetin-3-O-arabinoside	Flavonol	9	9	9	15186	12312	14205	10.593	1.675	up
mws0919	Kaempferol-3-O-rhamnoside (Afzelin)	Flavonol	9	9	9	10201	11946	10980	10.261	1.649	up
Hmcp002539	Isorhamnetin-3-O-rhamnoside	Flavonol	9	9	9	8634	10420	8992	10.021	1.629	up
pmp001309	6-Hydroxykaempferol-7-O-glucoside	Flavonol	17396	29554	11179	1139100	1057500	1068500	5.812	1.247	up
Lmyp004052	Quercetin-3-O-(6"-p-Coumaroyl) galactoside	Flavonol	33628	31329	33550	1430400	1257300	1350960	5.358	1.191	up
mws0059	Quercetin-3-O-rutinoside (Rutin)	Flavonol	162770	146930	144700	5719000	6083800	5890100	5.283	1.183	up
mws1290	Kaempferol-3-O-(6"-p-coumaroyl) glucoside (Tiliroside)	Flavonol	347570	303160	317330	5685900	4976000	5402300	4.053	1.036	up
Hmjp003123	Quercetin-5-O-glucuronide	Flavonol	9	9	9	9172000	8236800	8902500	19.894	2.296	up
pme0434	Procyanidin B2	Proanthocyanidins	9	9	9	56291	70259	62375	12.773	1.839	up
mws0836	Procyanidin B1	Proanthocyanidins	9	9	9	13794	10757	12543	10.424	1.661	up

Table S2. Differentially accumulated flavonoids between S2u and S2d.

Index	Compounds	Class	S2u-1	S2u-2	S2u-3	S2d-1	S2d-2	S2d-3	Log_2FC	VIP	Type
pmf0027	Cyanidin-3-O-galactoside	Anthocyanins	9	9	9	31798000	31682000	32052000	21.755	2.671	up
pmb0550	Cyanidin-3-O-glucoside	Anthocyanins	9	9	9	3424500	3863700	3658200	18.629	2.472	up
pme3392	Pelargonidin-3-O-glucoside	Anthocyanins	9	9	9	2237700	2140100	2210200	17.897	2.423	up
P4032	Cyanidin-O-rutinoside-O-malonylglucoside	Anthocyanins	9	9	9	1728000	1793100	1842400	17.600	2.403	up

P4329	Peonidin-O-rutinoside-O-malonylglucoside	Anthocyanins	9	9	9	241770	297100	275640	14.881	2.209	up
pmb0541	Cyanidin-3-O-(6"-O-malonyl)glucoside-5-O-glucoside	Anthocyanins	83932	88481	80336	30223000	38450000	35650000	8.689	1.687	up
pme1793	Pelargonidin-3,5-O-diglucoside	Anthocyanins	19674	19543	18184	1257200	1201200	1146500	5.973	1.400	up
HJN041	Epicatechin glucoside	Catechin derivatives	9	9	9	155130	168800	158920	14.126	2.152	up
pme0460	Epicatechin	Catechin derivatives	90166	112210	121400	2728200	2215400	2501800	4.523	1.218	up
mws0034	(-)-Epigallocatechin gallate	Catechin derivatives	21635	19238	14920	9	9	9	-11.013	1.899	down
mws0463	Hesperetin	Flavanone	9	9	9	6398	5933	7028	9.486	1.763	up
pmb0628	Eriodictyol-8-C-glucoside-4'-O-glucoside	Flavanone	72220	70206	64213	9	9	9	-12.902	2.057	down
mws0744	Dihydromyricetin	Flavanonol	160430	142150	143380	9017	11514	11028	-3.821	1.119	down
mws1661	Diosmin	Flavone	9	9	9	337560	306580	328650	15.137	2.228	up
pmb0592	Chrysoeriol-7-O-rutinoside-5-O-glucoside	Flavone	9	9	9	133650	109410	119860	13.714	2.120	up
pma6640	3'-O-Methyltricetin-7-O-glucoside	Flavone	117270	70130	90246	1008900	1431800	1188900	3.708	1.100	up
pmb0672	Apigenin-6-C-glucoside-7-O-(6"-feruloyl) glucoside	Flavone C-glycosides	9	9	9	111230	110400	119520	13.625	2.114	up
mws1608	Luteolin-6-C-glucoside	Flavone C-glycosides	3679	3107	3226	9	9	9	-8.535	1.672	down
Lmpp003268	Kaempferol-3-O-rutinoside-7-O-glucoside	Flavonol	9	9	9	5416700	4473000	4864850	19.060	2.500	up
Lmyn001269	Kaempferol-3-O-sophoroside	Flavonol	9	9	9	673810	630110	629850	16.128	2.300	up
Lmjp002867	Kaempferol-3-O-neohesperidoside	Flavonol	171990	58977	26426	9904800	9401300	9283600	6.795	1.520	up
mws0059	Quercetin-3-O-rutinoside (Rutin)	Flavonol	465540	386150	509840	5719000	6083800	5890100	3.700	1.102	up
Lmmp002963	6-C-Methylquercetin-3-O-rutinoside	Flavonol	9	9	9	57360	54295	58210	12.619	2.034	up
mws0988	Rhamnetin	Flavonol	89227	90534	90322	9	9	9	-13.288	2.088	down
mws1068	Kaempferol	Flavonol	191370	190570	188070	6636	4194	5230	-5.149	1.301	down
pme2954	Quercetin	Flavonol	5538700	5183400	5228800	681410	578770	640260	-3.069	1.003	down
Lmyp005328	Quercetin-3-O-(2"-cinnamoyl) Glucoside	Flavonol	432010	427450	453210	31445	19685	20565	-4.194	1.175	down
Lmhp002800	2'-Hydoxy,5-methoxyGenistein-4',7-O-diglucoside	Isoflavone	366290	530870	467170	5720000	5945200	6065600	3.700	1.102	up
mws0836	Procyanidin B1	Proanthocyanidins	9	9	9	13794	10757	12543	10.424	1.848	up
pme0436	Procyanidin B3	Proanthocyanidins	4596	10078	6647	167080	139460	149860	4.420	1.207	up
pme0434	Procyanidin B2	Proanthocyanidins	5816	7630	3750	56291	70259	62375	3.458	1.065	up

Table S3. Differentially accumulated flavonoids between S3u and S3d.

Index	Compounds	Class	S3u-1	S3u-2	S3u-3	S3d-1	S3d-2	S3d-3	Log_2FC	VIP	Type
pmb0550	Cyanidin-3-O-glucoside	Anthocyanins	9	9	9	4266400	3912600	4321900	18.821	2.649	up
P4032	Cyanidin-O-rutinoside-O-malonylglucoside	Anthocyanins	9	9	9	2859300	2562700	2673800	18.194	2.604	up
P4329	Peonidin-O-rutinoside-O-malonylglucoside	Anthocyanins	9	9	9	424090	508510	651720	15.841	2.428	up
pme3392	Pelargonidin-3-O-glucoside	Anthocyanins	9	9	9	3168900	3910100	3373200	18.562	2.619	up
pmb0541	Cyanidin-3-O-(6"-O-malonyl) glucoside-5-O-glucoside	Anthocyanins	71648	63492	59089	71929000	60711000	56457000	9.927	1.924	up
pmf0027	Cyanidin-3-O-galactoside	Anthocyanins	58694	58993	63096	35415000	32028000	27914000	9.043	1.836	up
pme1793	Pelargonidin-3,5-O-diglucoside	Anthocyanins	27499	30828	25174	2775000	2768800	2806000	6.644	1.574	up
HJN041	Epicatechin glucoside	Catechin derivatives	9	9	9	594910	597360	611930	16.028	2.445	up
pme0460	Epicatechin	Catechin derivatives	77830	58450	71342	4522600	4526400	4301600	6.007	1.497	up
mws0054	Catechin	Catechin derivatives	268560	241740	288240	2151300	2188100	1904900	2.967	1.051	up
pmb0628	Eriodictyol-8-C-glucoside-4'-O-glucoside	Flavanone	95931	64602	88042	9	9	9	-13.168	2.214	down
mws1661	Diosmin	Flavone	9	9	9	3678400	4684000	3371400	18.729	2.641	up
pmb0592	Chrysoeriol-7-O-rutinoside-5-O-glucoside	Flavone	9	9	9	331900	363440	493450	15.426	2.396	up
pmb0672	Apigenin-6-C-glucoside-7-O-(6"-feruloyl) glucoside	Flavone C-glycosides	9	9	9	291590	319520	306310	15.052	2.369	up
Lmjp002867	Kaempferol-3-O-neohesperidoside	Flavonol	9	9	9	61334000	69130000	63278000	22.775	2.914	up
Lmpp003268	Kaempferol-3-O-rutinoside-7-O-glucoside	Flavonol	9	9	9	13746000	12442000	14733000	20.531	2.767	up
Lmyn001269	Kaempferol-3-O-sophoroside	Flavonol	9	9	9	834070	862610	772030	16.480	2.479	up
Lmyp004318	Kaempferol-3-O-(6"-p-Coumaroyl) galactoside	Flavonol	665010	716580	641430	9	9	9	-16.193	2.457	down
Lmdp004267	Quercetin-3-O-(6"-p-Coumaroyl) glucoside	Flavonol	124020	143900	222140	9	9	9	-14.148	2.292	down
Lmsn002815	Kaempferol-3-O-rutinoside	Flavonol	22115	24047	19029	9	9	9	-11.237	2.046	down
Lmyp005328	Quercetin-3-O-(2"-cinnamoyl) glucoside	Flavonol	1318900	1420300	1342800	152210	144520	153960	-3.179	1.089	down
mws0836	Procyanidin B1	Proanthocyanidins	9	9	9	32586	33526	30297	11.802	2.098	up
pme0436	Procyanidin B3	Proanthocyanidins	5969	4962	6514	294960	289060	307770	5.676	1.455	up
pme0434	Procyanidin B2	Proanthocyanidins	8679	12434	10420	192600	174760	171310	4.094	1.236	up

Table S4. Differentially accumulated flavonoids between S4u and S4d.

Index	Compounds	Class	S4u-1	S4u-2	S4u-3	S4d-1	S4d-2	S4d-3	Log_2FC	VIP	Type
pmf0027	Cyanidin-3-O-galactoside	Anthocyanins	9	9	9	31856000	34635000	31909000	21.797	2.609	up
pmb0550	Cyanidin-3-O-glucoside	Anthocyanins	9	9	9	4084000	3938100	4234600	18.792	2.422	up
pme3392	Pelargonidin-3-O-glucoside	Anthocyanins	9	9	9	3552500	4508800	4163900	18.788	2.422	up
P4032	Cyanidin-O-rutinoside-O-malonylglucoside	Anthocyanins	9	9	9	2337800	2763300	2950000	18.186	2.383	up
P4329	Peonidin-O-rutinoside-O-malonylglucoside	Anthocyanins	9	9	9	415060	301820	306510	15.210	2.178	up
pmb0541	Cyanidin-3-O-(6''-O-malonyl) glucoside-5-O-glucoside	Anthocyanins	62923	45400	42775	37900000	36958000	31467000	9.459	1.720	up
pme1793	Pelargonidin-3,5-O-diglucoside	Anthocyanins	22466	21082	22129	2631800	2868400	2676700	6.960	1.474	up
HJN041	Epicatechin glucoside	Catechin derivatives	9	9	9	594570	592650	583960	16.001	2.235	up
pme0460	Epicatechin	Catechin derivatives	26905	24890	24916	3235500	3181300	2947700	6.932	1.471	up
mws0054	Catechin	Catechin derivatives	150760	147320	140080	1648900	1734700	1609200	3.510	1.047	up
mws0057	Eriodictyol 7-O-glucoside	Flavanone	87136	126450	104500	9	9	9	-13.524	2.053	down
pmb0628	Eriodictyol-8-C-glucoside-4'-O-glucoside	Flavanone	55072	65933	62355	9	9	9	-12.729	1.993	down
mws1661	Diosmin	Flavone	9	9	9	3616000	3724200	3540800	18.620	2.411	up
pmb0592	Chrysoeriol-7-O-rutinoside-5-O-glucoside	Flavone	9	9	9	482210	429320	390490	15.557	2.204	up
HJN079	Pinocembrin-7-O-rutinoside	Flavone	17473	15165	18007	9	9	9	-10.873	1.842	down
pmp000115	Monohydroxy-hexamethoxyflavone	Flavone	16228	21198	11805	9	9	9	-10.832	1.835	down
Lmjp005224	4',5,7-Trihydroxy-3',3,6-Trimethoxyflavone	Flavone	5166	8261	4297	9	9	9	-9.358	1.703	down
pmp000004	4',5,7-Trihydroxy-3',6-dimethoxyflavone	Flavone	1770	4288	2453	9	9	9	-8.300	1.597	down
Zmhp004269	Skullcapflavone II	Flavone	12994	20114	13304	742	2885	1221	-3.259	1.010	down
pmb0672	Apigenin-6-C-glucoside-7-O-(6''-feruloyl) glucoside	Flavone C-glycosides	9	9	9	446600	350350	422880	15.463	2.197	up
Lmjp002867	Kaempferol-3-O-neohesperidoside	Flavonol	9	9	9	75154000	74620000	76817000	23.001	2.680	up
Lmpp003268	Kaempferol-3-O-rutinoside-7-O-glucoside	Flavonol	9	9	9	14299000	12173000	14185000	20.522	2.531	up
Lmyn001269	Kaempferol-3-O-sophoroside	Flavonol	9	9	9	720580	964720	787060	16.483	2.265	up
Lmsn002815	Kaempferol-3-O-rutinoside	Flavonol	18424	15872	16746	9	9	9	-10.885	1.843	down

Lmyp005328	Quercetin-3-O-(2''-cinnamoyl) glucoside	Flavonol	864580	1022900	927840	42031	46534	47335	-4.373	1.168	down
mws0836	Procyanidin B1	Proanthocyanidins	9	9	9	33399	32434	32555	11.831	1.922	up
pme0436	Procyanidin B3	Proanthocyanidins	6883	6370	7406	289070	257690	263390	5.293	1.286	up
pme0434	Procyanidin B2	Proanthocyanidins	7965	14027	9825	247020	260620	241440	4.557	1.195	up

Table S5. List of 17 common compounds that were identified in four comparisons from S1 to S4.

Index	Compounds	Class	type
P4032	Cyanidin-O-rutinoside-O-malonylglucoside	Anthocyanins	up
P4329	Peonidin-O-rutinoside-O-malonylglucoside	Anthocyanins	up
pmb0541	Cyanidin-3-O-(6''-O-malonyl) glucoside-5-O-glucoside	Anthocyanins	up
pmb0550	Cyanidin-3-O-glucoside	Anthocyanins	up
pme1793	Pelargonidin-3,5-O-diglucoside	Anthocyanins	up
pme3392	Pelargonidin-3-O-glucoside	Anthocyanins	up
pmf0027	Cyanidin-3-O-galactoside	Anthocyanins	up
HJN041	Epicatechin glucoside	Catechin derivatives	up
pmb0628	Eriodictyol-8-C-glucoside-4'-O-glucoside	Flavanone	down
mws1661	Diosmin	Flavone	up
pmb0592	Chrysoeriol-7-O-rutinoside-5-O-glucoside	Flavone	up
pmb0672	Apigenin-6-C-glucoside-7-O-(6''-feruloyl) glucoside	Flavone C-glycosides	up
Lmjp002867	Kaempferol-3-O-neohesperidoside	Flavonol	up
Lmpp003268	Kaempferol-3-O-rutinoside-7-O-glucoside	Flavonol	up
Lmyn001269	Kaempferol-3-O-sophoroside	Flavonol	up
mws0836	Procyanidin B1	Proanthocyanidins	up
pme0434	Procyanidin B2	Proanthocyanidins	up

Table S6. Carotenoids found in the blotch and non-blotch petal parts of *R. persica* at different stages of flower development (μg·g^{-1}).

sample	phytoene	β-carotene	β-cryptoxanthin	lutein	neoxanthin	zeaxanthin	violaxanthin	antheraxanthin	α-cryptoxanthin	γ-carotene	capsanthin	ε-carotene	lycopene	β-apocarotenal
S1-1	1.290	21.800	1.640	130.000	9.650	8.270	7.990	NA	0.230	0.096	0.002	NA	NA	0.275
S1-2	1.180	21.800	1.830	131.000	9.690	8.570	6.570	NA	0.216	0.081	0.002	NA	NA	0.272
S1-3	1.080	21.400	1.550	132.000	9.410	8.590	7.030	NA	0.242	0.083	0.002	NA	NA	0.277
S2d-1	18.100	21.900	2.530	138.000	10.500	16.100	35.700	7.090	0.100	0.098	0.026	NA	0.104	0.279
S2d-2	17.528	21.870	2.354	143.727	10.847	17.122	37.902	7.219	0.104	0.097	0.027	NA	0.115	0.283
S2d-3	18.440	22.375	2.745	139.444	10.943	15.799	35.161	6.892	0.100	0.094	0.025	NA	0.109	0.281
S2u-1	55.700	26.400	3.300	231.000	18.100	116.000	45.900	26.600	0.352	0.203	0.095	NA	0.125	0.282
S2u-2	63.400	30.000	3.780	262.000	20.100	138.000	52.700	29.500	0.384	0.215	0.124	NA	0.140	0.291
S2u-3	60.700	26.700	3.450	246.000	21.200	128.000	42.300	19.600	0.359	0.206	0.120	NA	0.168	0.279
S3d-1	201.000	4.720	2.300	55.700	5.690	35.000	86.400	21.700	0.114	0.293	0.053	0.032	0.199	0.276
S3d-2	204.000	4.830	2.260	59.100	5.370	35.900	86.200	22.400	0.110	0.287	0.054	0.042	0.196	0.279
S3d-3	212.000	4.600	2.380	56.700	5.630	28.500	86.500	21.800	0.107	0.277	0.045	0.039	0.187	0.275
S3u-1	3150.000	9.730	3.880	96.300	15.900	255.000	30.700	115.000	0.280	1.640	0.197	0.481	0.643	0.295
S3u-2	3320.000	8.910	4.110	98.600	12.600	279.000	26.700	107.000	0.332	1.360	0.196	0.460	0.623	0.308
S3u-3	3520.000	9.250	4.090	104.000	13.700	293.000	25.600	108.000	0.311	1.320	0.228	0.498	0.667	0.303
S4d-1	195.000	3.370	2.030	56.700	5.940	42.100	93.900	13.300	0.096	0.226	0.040	0.027	0.174	0.270
S4d-2	186.000	3.340	1.950	56.000	5.820	41.800	83.600	14.200	0.108	0.258	0.044	0.032	0.176	0.282
S4d-3	203.000	3.220	2.130	56.500	4.930	40.000	85.300	14.000	0.093	0.268	0.042	0.022	0.169	0.278
S4u-1	3670.000	8.820	4.280	113.000	12.200	226.000	97.800	91.600	0.271	1.310	0.165	0.519	0.815	0.328
S4u-2	3380.000	8.930	4.310	112.000	13.100	229.000	84.300	89.300	0.313	1.160	0.157	0.507	0.746	0.332
S4u-3	3340.000	8.460	4.830	113.000	13.500	228.000	110.000	87.800	0.307	1.340	0.143	0.436	0.770	0.332

Table S7. The quality analysis of all reads from 21 samples.

Sample	Raw Reads	Clean Reads	Q20 (%)	Q30 (%)	GC content (%)	Unique mapped (%)	Total mapped (%)
S1-1	48,864,716	48,784,312	97.88	93.85	47.92	89.80	91.52
S1-2	51,725,866	51,645,294	97.97	94.06	47.54	94.55	96.29
S1-3	48,906,146	48,828,742	97.85	93.81	47.92	94.17	95.84
S2d-1	50,271,726	50,189,752	98.00	94.16	47.46	93.75	95.48
S2d-2	52,489,208	52,398,068	97.99	94.12	47.36	94.65	96.39
S2d-3	52,210,140	52,127,586	98.12	94.45	47.48	93.92	95.65
S2u-1	45,593,070	45,512,664	97.92	93.96	47.24	93.85	95.64
S2u-2	52,449,452	52,375,070	98.05	94.27	47.13	94.25	96.11
S2u-3	57,340,612	57,227,586	98.00	94.22	47.32	94.87	96.74
S3d-1	55,539,362	55,449,576	98.24	94.68	47.29	93.86	95.81
S3d-2	52,698,274	52,610,422	98.02	94.21	47.02	93.59	95.47
S3d-3	46,811,072	46,746,760	98.07	94.26	47.29	93.86	95.74
S3u-1	44,697,182	44,628,268	98.06	94.24	46.98	93.94	96.03
S3u-2	41,540,316	41,466,920	97.97	94.03	46.68	92.53	94.65
S3u-3	47,802,558	47,715,316	97.49	92.85	46.70	93.42	95.52
S4d-1	43,514,304	43,447,968	98.01	94.17	47.32	93.74	96.14
S4d-2	45,281,740	45,205,406	97.99	94.14	48.12	92.41	94.68
S4d-3	43,759,148	43,687,222	98.02	94.19	47.68	92.60	94.91
S4u-1	39,959,482	39,898,184	98.17	94.55	47.42	93.60	96.32
S4u-2	43,497,460	43,416,262	97.97	94.09	47.31	93.94	96.64
S4u-3	48,858,026	48,749,510	98.02	94.21	48.41	93.26	95.67

Table S8. Correlation analysis of seven anthocyanins and flavonoid-related DEGs.

Anthocyanins	DEGs	Correlation	*P*-Value
pmb0541	Rbe016466	0.942	0.002
pmb0541	Rbe028518	0.926	0.003
pmf0027	Rbe026328	0.888	0.008
pmb0550	Rbe016466	0.863	0.012
pmf0027	Rbe016466	0.858	0.013
pmb0550	Rbe026328	0.854	0.014
pmf0027	Rbe013916	0.806	0.028
pme1793	Rbe028518	0.800	0.031
pme3392	Rbe026328	0.773	0.041
pmb0550	Rbe013916	0.773	0.041
pmb0541	Rbe014123	0.769	0.043
pmb0550	Rbe028518	0.768	0.044
pme1793	Rbe016466	0.763	0.046
pmf0027	Rbe014123	0.757	0.049
pme3392	Rbe016466	0.753	0.051

Table S9. Transcription factors identified in the cyan and saddlebrown modules.

GeneID	Module	Symbol	TF family	Description
Rbe024976	cyan	NAC100	NAC	NAC domain containing protein 50-like [Rosa chinensis]
Rbe009491	cyan	MYB4	MYB_related	transcription factor MYB4-like [Rosa chinensis]
Rbe009787	cyan	ZAT9	C2H2	zinc finger protein 197 [Prunus persica]
Rbe015696	cyan	NAC083	NAC	NAC domain-containing protein 83-like [Rosa chinensis]
Rbe006355	cyan	HHO5	G2-like	transcription factor HHO5-like isoform X1 [Rosa chinensis]
Rbe028685	cyan	WIP2	C2H2	zinc finger protein WIP2 [Rosa chinensis]

Rbe010942	cyan	MYB73	MYB	transcription factor MYB44-like [Rosa chinensis]
Rbe009701	cyan	MADS2	MADS	developmental protein SEPALLATA 1-like isoform X1 [Rosa chinensis]
Rbe005681	cyan	MYB123	MYB	anthocyanin regulatory C1 protein-like [Rosa chinensis]
Rbe024880	cyan	MYB1	MYB	MYB5 [Rosa rugosa]
Rbe006322	cyan	DOF5.3	DOF	dof zinc finger protein DOF3.4-like [Rosa chinensis]
Rbe021770	cyan	MYB62	MYB	transcription factor MYB24 [Rosa chinensis]
Rbe009935	cyan	ATHB-20	HD-ZIP	homeobox-leucine zipper protein HAT7 [Rosa chinensis]
MSTRG.22677	cyan	MYB3	MYB3	transcription factor MYB3-like [Rosa chinensis]
Rbe014352	cyan	MYB73	MYB	transcription factor MYB44-like [Rosa chinensis]
Rbe014309	cyan	GATA12	GATA	GATA transcription factor 12 [Rosa chinensis]
Rbe028610	cyan	PHL5	G2-like	myb family transcription factor PHL5 isoform X1 [Rosa chinensis]
Rbe015934	cyan	MYB86	MYB	transcription factor MYB86-like [Rosa chinensis]
Rbe002625	cyan	WIP3	C2H2	zinc finger protein WIP3 [Rosa chinensis]
Rbe003454	cyan	BLH8	TALE	BEL1-like homeodomain protein 8 [Rosa chinensis]
Rbe027753	cyan	WDR44	WDR44	WD repeat-containing protein 44-like [Rosa chinensis]
Rbe006154	cyan	RL3	MYB_related	PREDICTED: protein RADIALIS-like 4 [Fragaria vesca subsp. vesca] [Fragaria vesca]
Rbe008908	saddlebrown	MADS1	MADS	floral homeotic protein AGAMOUS isoform X2 [Rosa chinensis]
Rbe013941	saddlebrown	BLH3	TALE	BEL1-like homeodomain protein 3 isoform X2 [Rosa chinensis]
Rbe021137	saddlebrown	BHLH147	bHLH	transcription factor bHLH147-like [Rosa chinensis]
Rbe024860	saddlebrown	ERF023	ERF	ethylene-responsive transcription factor ERF023 isoform X2 [Rosa chinensis]
Rbe026320	saddlebrown	VRN1	B3	B3 domain-containing transcription factor VRN1-like [Rosa chinensis]

Table S10. Correlation analysis of seven anthocyanins and transcription factors in the cyan module.

Anthocyanins	DEGs	Correlation	*P*-Value
pmb0541	Rbe003454	0.967	0.000
pmf0027	Rbe021770	0.939	0.002
pmb0541	Rbe021770	0.938	0.002

pmb0550	Rbe021770	0.935	0.002
pmb0541	Rbe015934	0.928	0.003
pmb0541	Rbe028610	0.919	0.003
pmb0541	Rbe027753	0.908	0.005
pmf0027	Rbe009701	0.906	0.005
pmb0550	Rbe009701	0.882	0.009
pme1793	Rbe003454	0.879	0.009
pmb0541	MSTRG.22677	0.877	0.010
pmb0541	Rbe009935	0.862	0.013
pmb0541	Rbe006154	0.855	0.014
pmf0027	Rbe006154	0.851	0.015
pmb0550	Rbe006154	0.838	0.019
pmb0541	Rbe002625	0.837	0.019
pme3392	Rbe021770	0.837	0.019
pmb0550	Rbe003454	0.837	0.019
P4329	MSTRG.22677	0.831	0.020
pme3392	Rbe003454	0.820	0.024
pme1793	Rbe021770	0.817	0.025
pmb0550	Rbe028610	0.809	0.027
pmb0550	Rbe015934	0.805	0.029
pmb0541	Rbe009701	0.803	0.030
pmf0027	Rbe003454	0.803	0.030

Table S11. Primers used for the qRT-PCR analysis.

Gene ID	Gene name	Forward primer (5' to 3')	Reverse primer (5' to 3')
Reference gene	*GAPDH*	GGTCAAGGTCATTGCTTGGT	GGATCGATCACATCGACAGA
Rbe013916	*DFR*	TCTCCGATCATCACCTCTATCCTTA	TGCCTTCCACAGCGTCAA

Rbe016466	*ANS*	CCCAAAGAGGAGCTCATTAACA	TGATGTCCTCGGAGTCTATCTC
Rbe26328	*UFGT*	TGTTGTTGTCGGAGATGGCT	GAGACCACAAGAATGGAACCC
Rbe028518	*4CL*	TCGGTATCTTACTCGGAACTCT	CCAAGAACACAACGGGATAGT
Rbe014123	*4CL*	TCCTCTCTCCCAACTCAATCT	TCGAATCTGCGACCTGTTTAG
MSTRG.22677	*MYB*	GGTAAGAGTTGTCGACTGAGATG	CACCTATTCCCAAGGAGTGTATG
Rbe009701	*MADS2*	CTGAGGTTGCTCTCATCATCTT	TTGACTTCCACTGCACCATAG
Rbe009935	*HDZIP*	AAGCAGCTGGAGAAAGACTATG	CAGCCTGAAGTTTCTTGTTGTG
